# ID 178 - Métodos de Avaliação para Incorporação OPME no SUS: um protocolo de direcionamento

**DOI:** 10.5327/2237-9622.2025.v34s1.178

**Published:** 2025-11-25

**Authors:** Kátia Elizabete Galdino, Ketinlly Yasmyne Nascimento Martins, Anna Kellssya Leite Filgueira, Bárbara Sousa dos Santos, Mônica Vinhas de Sousa, Rodolfo Ramos Castelo Branco, Eduardo Jorge Valadares Oliveira

**Keywords:** avaliação da tecnologia biomédica, tecnologia assistiva, aparelhos ortopédicos

## Abstract

**Introdução:**

Com foco em fortalecer o processo de gestão de tecnologias no âmbito do SUS, a Política Nacional de Gestão de Tecnologias em Saúde (PNGTS), incentiva a utilização de evidências científicas no subsídio para incorporação.

No campo de órteses, próteses e materiais especiais (OPME), a ATS apresenta limitações específicas e exige, portanto, uma visão particular somada, com frequência, à necessidade de adaptações, revisões ou construção de processos diversos, que tenham maior especificidade. Desse modo, este estudo tem por finalidade construir um guia para descrever os métodos de avaliação para incorporação de OPME no SUS, ressaltando as particularidades que envolvem essa classe de dispositivos e a literatura acerca dela.

**Método:**

Três etapas metodológicas foram estabelecidas:

Na primeira etapa foram mapeados e identificados os critérios relevantes para síntese de evidências clínicas e científicas sobre OPME. A definição desses critérios foi marcada por conversa com especialistas associada ao reconhecimento dos processos de prescrição, concessão e adaptação já realizados em oficinas ortopédicas de referência e em Centros Especializados em Reabilitação (CERs) em diversas regiões do Brasil.

Na segunda etapa, foi realizada uma análise comparativa entre as etapas formais de condução de uma ATS e as particularidades de uma avaliação de OPME para que houvesse identificação dos tópicos ajustáveis visando atender às especificidades e delineando as fases desse processo.

Com as informações obtidas nas etapas anteriores, a etapa 3 destinou-se à elaboração do G*uia de Boas Práticas em Avaliação de* OPME.

**Resultados:**

A busca por potenciais estudos elegíveis deve ser ampla, sensível e sistemática a fim de identificar todas as evidências científicas disponíveis. A estratégia de busca, ao se tratar de OPME, deve ser estruturada com base nos elementos da pergunta de pesquisa, assim como a condução padrão de ATS, no entanto, devido à ampla gama de termo técnico na área, será necessário, em muitas ocasiões, a inclusão de termos associados ao vocabulário não controlado, assim necessitando uma busca prévia na literatura.

Outra particularidade é a possibilidade de, por ausência de especificidade, o instrumento de avaliação migrar de revisão sistemática para revisão de escopo. A decisão entre os tipos de estudo deve ser guiada pelos objetivos da pesquisa, pela natureza do tema e/ou pela condição da literatura existente. A síntese de evidências nessa área de atuação também poderá ser de duas formas: qualitativas e/ou quantitativas. A análise qualitativa visa explorar questões sobre experiências, percepções, ou significados atribuídos pelos participantes. Já a análise quantitativa, por sua vez, está voltada para a quantificação de efeitos ou associações, como a eficácia de uma intervenção.

**Conclusão:**

As OPMEs possuem características particulares que devem ser consideradas na realização de estudos de ATS, visando a um processo final coerente e eficiente que permita aos gestores avaliar a relevância relativa dos diferentes domínios ou estágios de interesse, bem como o desempenho da referida tecnologia em comparação com alternativas. Essas singularidades exigem uma abordagem sistemática e analítica, baseada em fundamentos de alta qualidade, com intervalos claros e predefinidos que foram sintetizados na construção deste guia com o intuito de aumentar a transparência e a reprodutibilidade do processo decisório, garantindo que os pacientes atendidos pelo SUS recebam os melhores resultados.

